# Physiological Activity of Neuropeptide F on the Hindgut of the Blood-Feeding Hemipteran, *Rhodnius prolixus*

**DOI:** 10.1673/031.009.5701

**Published:** 2009-07-15

**Authors:** Ronald Gonzalez, Ian Orchard

**Affiliations:** ^1^ Department of Biology, York University, 4700 Keele St., Toronto, ON, Canada M3J IP3; ^2^ Department of Biology, University of Toronto Mississauga, 3359 Mississauga Rd, Mississauga, ON, L5L 1C6

**Keywords:** insect, neuropeptide Y, myoinhibitory

## Abstract

Current hypotheses propose that, in the invertebrates, neuropeptide F (NPF), the vertebrate NPY homologue, may be capable of regulating responses to diverse cues related to nutritional status and feeding. An investigation into the effects of *Drosophila melanogaster* NPF (DrmNPF) and *Anopheles gambiae* NPF (AngNPF) on hindgut physiology of *Rhodnius prolixus* Stal (Heimptera: Reduviidae) suggests a myoinhibitory role for these peptides and the *R. prolixus* native peptide. Extracts of the central nervous system of *R. prolixus* were processed and several HPLC-fractions revealed NPF-like activity within the nanomolar equivalent range when tested using the hindgut contraction assay. Although NPF has been shown to decrease epithelial membrane potential in *Aedes aegypti* larval midgut preparations, NPF does not appear to play a role in epithelial transport of potassium in the hindgut. While the function of NPF has yet to be established, NPF-like effects suggest multiple physiological roles for NPF among invertebrates.

## Introduction

Since the identification of neuropeptide F (NPF) in the sheep tapeworm, *Moniezia expansa* (Maule et al. 1992), and the discovery of this family in other invertebrates, there has been debate as to its physiological role. Hypotheses about the function of NPF were originally developed based on the distribution of NPF-like immunoreactivity. For instance, the digestive systems of platyhelminthes contain many NPF-like immunoreactive processes, especially in the esophagus. Similarly, turbellarians, with either a muscular pharynx or an esophagus leading into a blind intestine, are all found to contain NPF-like immunoreactive processes and cell bodies (Maule and Day 1999). In addition, NPF-like immunoreactivity in helminth parasites is also found within the reproductive system, especially in the egg-forming chamber, indicating a potential role for the peptide in egg-formation (Halton et al. 1994). The immunoreactive staining in flatworms suggests a possible role for NPF in the coordination of reproductive processes, somatic musculature, organs of attachment, and digestion. However, these hypotheses have been difficult to test due to lack of bioassays for any of these specific functions in flatworms.

Regulation of the digestive tract in response to feeding and its integrative role in whole-animal homeostasis are only known in general terms (Onken et al. 2004). The regulatory inputs that act upon this system are believed to involve neuroendocrine factors from the central nervous system (CNS), or from the numerous enteroendocrine cells expressing one or more peptide hormones (Brown et al. 1985). In insects NPF-like immunoreactivity has also been shown to be associated with the digestive system. In the dipterans, *Drosophila melanogaster* and *Aedes aegypti,* NPF-like immunoreactive endocrine cells are present in larval midgut (Brown *et al.* 1999; Stanek et al. 2002). However, the larval gut of *A. aegypti* appears to contain a ring of NPF-like immunoreactive cells at the junction between the fore- and midgut. It has been proposed in *A. aegypti,* that NPF may play a role in the regulation of matrix secretion in larvae (Stanek et al. 2002). In *Rhodnius prolixus* we have recently shown the distribution of NPF-like immunoreactive processes present over the surface of the hindgut and the immunoreactivity in these processes is greatly reduced in intensity 24 hours post-feeding (Gonzalez and Orchard 2008).

In dipteran insects, a role for NPF in the coordination of physiological processes dependent on nutritional status has been suggested in *A. aegypti* and *D. melanogaster.* For example, in *A. aegypti,* the hemolymph titer of *Aedes* NPF (AeaNPF) changes in adult females shortly after a blood meal, suggestive of a link to digestion and reproduction (Stanek et al. 2002). Additionally, *A. aegypti* larvae have decreased stomach motility and transepithelial voltage in response to submicromolar applications of AeaNPF (Onken et al. 2004). Research in *D. melanogaster* has established that the NPF neuronal circuit responds to chemosensory stimuli of sugar, and promotes the feeding response (Shen and Cai 2001).

The neuropeptide Y family has been more thoroughly examined among vertebrate species although there are still significant gaps in our understanding of the specific functions attributed to NPF or NPY. One of the major obstacles to determining NPF function in insects has been a general lack of tissue or organ-based bioassays (McViegh et al. 2005). In contrast, vertebrate assays present a considerable confounding impediment because of the overlapping and counteracting roles of various Y receptors. Therefore, the study of an insect model such as *R. prolixus,* with its interesting feeding activities, presents an opportunity to investigate some of the possible physiological effects of NPF. *R. prolixus* is an obligate blood-feeder that requires gorging on blood, interspersed with no feeding, in order to survive. Due to these large periods in which there is an absence of feeding and therefore a stable baseline, *R. prolixus* lends itself to the study of neuropeptides involved in regulating feeding, such as NPF.

## Material and Methods

### Animals

Fifth instars of *R. prolixus* Stal (Heimptera: Reduviidae) were used from a long standing colony, maintained at 25°C under high humidity in a cycle of 12:12 (L:D). Unless indicated, the insects were 6–8 weeks post-ecdysis having previously fed on rabbit blood as 4^th^ instars. Hindgut tissues were taken from unfed male and female fifth instars.

### Solutions and chemicals

The physiological saline used was based on *R. prolixus* hemolymph ion composition (Lane et al. 1975) and consisted of: NaCl, 150mM; KCl, 8.6mM, CaCl_2_, 2.OmM, NaHCO_3_, 4.0mM; MgCl_2_, 8.5mM; Hepes, 5.0mM, Glucose, 34mM. The pH was adjusted to 7.0 with NaOH. The above components, in addition to *Leucophaea* leucokinin 1 (LK 1), were purchased from Sigma-Aldrich (www.sigmaaldrich.com). *D. melanogaster* neuropeptide F (DrmNPF) and *Anopheles gambiae* neuropeptide F (AngNPF) were provided by Dr M. R. Brown (University of Georgia, Athens, USA). Stock solutions of peptides were stored at -20°C before their use. NPF-like radioimmunoassay (RIA) positive fractions of interest (see Gonzalez and Orchard 2008) were thawed, sub-sampled, and dried in a Speed-Vac concentrator (Savant, www.combichemlab.com) and then reconstituted in physiological saline using the absolute amounts of NPF-like peptide as determined from NPF RIA standard curve.

### Hindgut contraction assay

The hindguts of 5^th^ instar *R. prolixus* were dissected out in physiological saline along with a small portion of the ventral cuticle surrounding the anus and a small portion of the posterior midgut. The cuticle was secured to a Sylgard-coated (Dow-Corning, ww.corning.com/lifesciences) dissecting dish using minuten pins, and the remaining midgut was attached to a miniature force transducer (Aksjeselskapet Mikroelectronikk, www.osioptoelectronics.no/) using a strand of hair. Changes in the frequency of muscle contractions and changes in basal tonus were recorded using BIOPAC MP100 System hardware and AcqKnowledge Version 3.5 software (BIOPAC Systems, Inc., www.biopac.com). Hindgut preparations were equilibrated in 100 µl of saline for 20 minutes, with repeated saline washes in 5 minute intervals. Subsequently, saline or peptide was applied to the preparation and hindgut activity was monitored for 5 minutes. This was followed by 20 minutes of saline washes, in 5 minute intervals, before repeating with test solutions containing peptides. The tissues were then allowed to recover for 20 minutes, with saline washes at 5 minute intervals, and recordings were conducted to ensure hindgut activity had not deteriorated. Peptides were reconstituted in double-distilled water to yield a stock solution of 1 mM peptide. Working dilutions were made up in saline from frozen aliquots of the stock solution. Peptides were applied by replacing the volume of saline bathing the preparation with the desired concentration of peptide in fresh saline. Data collected from the AcqKnowledge software was imported into Clampfit Version 10.0 (Molecular Devices Corporation, www.moleculardevices.com) software for analysis. The first minute of recordings was not included in the analyses to avoid the mechanical artefacts resulting from the initial application of test solutions; only the last 4 minutes of the 5 minute trials were analyzed. Measurements of frequency and average amplitude of hindgut contractions were calculated. To calculate frequency and amplitude of hindgut contractions, a threshold detection limit search using the Clampfit program was used. A baseline was set which corresponded to the baseline tension of the preparation. A re-arm marker was also set which corresponded to ∼50% average amplitude for all contractions within the measured time period in each trial. Any change in force which crossed the re-arm marker twice (both up and down) was counted as one contraction. The height of each contraction within the analyzed time period was averaged for measurements of amplitude, and the number of contractions detected was divided by the length of time for each trial for measurements of frequency. Each dose-response curve was fitted with a sigmoidal dose-response curve constructed using GraphPad Prism 4.0 (GraphPad Software, www.graphpad.com). For the fitted sigmoidal dose-response curve, max and min value parameters were set to 100 and 0, respectively.

### Reverse phase high-performance liquid chromatography (RP-HPLC)

Central nervous tissues from 225 unfed *R. prolixus* fifth instars were prepared for fractionation by a RP-HPLC equipped Brownlee C_18_ column with an 80Å pore size (Alltech/Mandel Scientific, www.mandel.ca) as has been previously described in detail (Gonzalez and Orchard 2008). Fractions (1 ml) were collected and subsamples of fractions were dried in a Speed-Vac concentrator and frozen at -20°C. Samples were assayed in a DrmNPF radioimmunoassay (see Gonzalez and Orchard 2008). Previous immunoreactivity obtained using the NPF-like RIA guided the physiological testing of fractions of interest (Gonzalez and Orchard 2008). NPF-like RIA positive fraction 32 from the C_18_ column in RP-HPLC was tested in the hindgut contraction assay. For further purification of NPF-like material, fractions 31, 32, and 33 were pooled. The pooled fractions were dried down, reconstituted and subsequently fractionated by RP-HPLC equipped with a phenyl column (Altech/Perkin Elmer, www.perkinelmer.com). Pooled fractions (31–33) from the C_18_ column were run through a phenyl column in RP-HPLC. NPF-like RIA positive fractions detected were found between fractions 50–55 and these fractions were tested in the hindgut contraction assay for bioactivity of the putative native NPF. Peptides within the dried fractions were dissolved in saline and applied to the hindgut preparation as previously described. One-way ANOVA with Newman-Keuls post test was performed using GraphPad Prism version 4.00. Measurements are expressed with their standard error of the mean (SEM).

### Scanning ion-selective electrode technique (SIET) measurements

An *in vitro* assay for the measurement of K^+^ concentration gradients near the surface of hindguts of 5^th^ instar *R prolixus* using the SIET (formerly referred to as SeRIS) was developed. Single hindguts were dissected in physiological saline by cutting the posterior end of the hindgut away from the ventral cuticle and keeping a small portion of the posterior midgut intact. The hindgut was secured to the base of a Sylgard-coated (Dow-Corning) dish by inserting a single minuten pin into the posterior midgut and positioning a rectangular glass slide (3mm × 5mm × 2mm) over the posterior aspect of the hindgut. The glass slide was held firm using a pin attached to a magnetic base. The hindgut was placed in the dish so that the dorsal aspect was available for measurement of K^+^ concentration gradients. Preparations were maintained in 1 ml of saline at room temperature. Application of peptide or drug was conducted by removing 500 µl of the saline and adding 500 µl of saline containing twice the desired concentration of peptide or drug.

The construction of microelectrodes for use with the SIET has been previously described in detail ([Bibr bibr18][Bibr bibr19]; Donini and O'Donnell 2005). Briefly, K^+^ selective microelectrodes were fabricated from 1.5 mm, non-filamented glass capillary tubes which were pulled to tip diameters of ∼5 µm, exposed to *N,N-*dimethyltrimethylsilylamine vapours and backfilled with 500 mM KCl. The microelectrode was subsequently front-filled with a 250–300 µm column length of K^+^ ionophore I cocktail B (Fluka, www.fluka.com). The SIET system and protocol employed in this study is described in detail in Rheault and O'Donnell (2004), with the following modifications. The K^+^ concentration gradients were measured over excursion distances of 100 µm and final K^+^ fluxes were calculated by subtracting the noise measured at a reference position 2 mm from the preparation from the signal measured adjacent to the preparation. The typical signals were 175 µV and typical noise was 20 µV. The ‘wait’ and ‘sample’ periods were 2 and 1 s, respectively, and the fluxes were reported as an average of 3 repetitive measurements at each site. SIET microelectrodes were calibrated in 150 mM KCl and 15 mM KCl plus 135 mM NaCl solutions. Microelectrode slopes for a tenfold change in ion concentration was 52.2 “ 0.60 mV. Calibration of electrodes and data collection was conducted using ASET 2.0 software (Science Wares Inc., www.sciencewares.com).

### Calculation of ion fluxes

Voltage differences were converted into concentration gradients using the following equation:

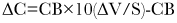

Where the *ΔC* is the concentration gradient between the two points measured in µmol/L·cm^3^ ; *C_B_* is the background ion concentration, calculated as the average of the concentration at each point measured in µmol/L; Δ*V* is the voltage gradient obtained from ASET in µV; and *S* is the slope of the electrode (mV). For ion-selective microelectrodes, measurements are made in terms of ion activity, not concentration. However, if the ion activity coefficient is the same in the calibration and experimental solutions, the data can be expressed in terms of concentrations. The concentration gradient can be subsequently converted into flux using Fick's law of diffusion in the following equation:

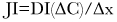

Where *J_I_* is the net flux of the ion in pmol/cm^2^ ·s; *D_I_* is the diffusion coefficient of the ion (1.19 × 10^-5^ cm^2^/s); *ΔC* is the concentration gradient in pmol/cm^3^ ; and *Δx* is the distance between the two points measured in cm. Results are expressed as means of the 3 repetitive measurements and their associated standard error (SEM).

## Results

### Effects of DrmNPF and AngNPF on spontaneous and leucokinin 1-induced hindgut contractions

**Figure 1.**
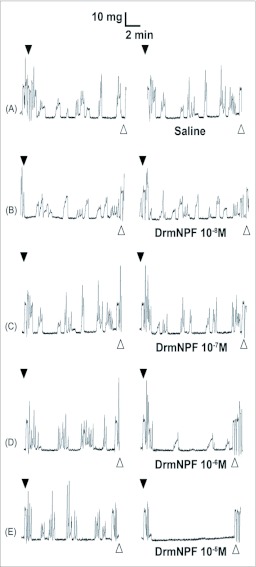
Effects of increasing concentrations of DrmNPF on spontaneous contractions of a single isolated *Rhodnius prolixus* hindgut. Closed arrows indicate addition of saline (left column) or various concentrations of DrmNPF (right column). The addition of DrmNPF was preceded by recordings of spontaneous contractions alone (A–E). Open arrows indicate washing with physiological saline. The preparation was washed at 5 minute intervals for 20 minutes between applications of peptide solutions.

**Figure 2.**
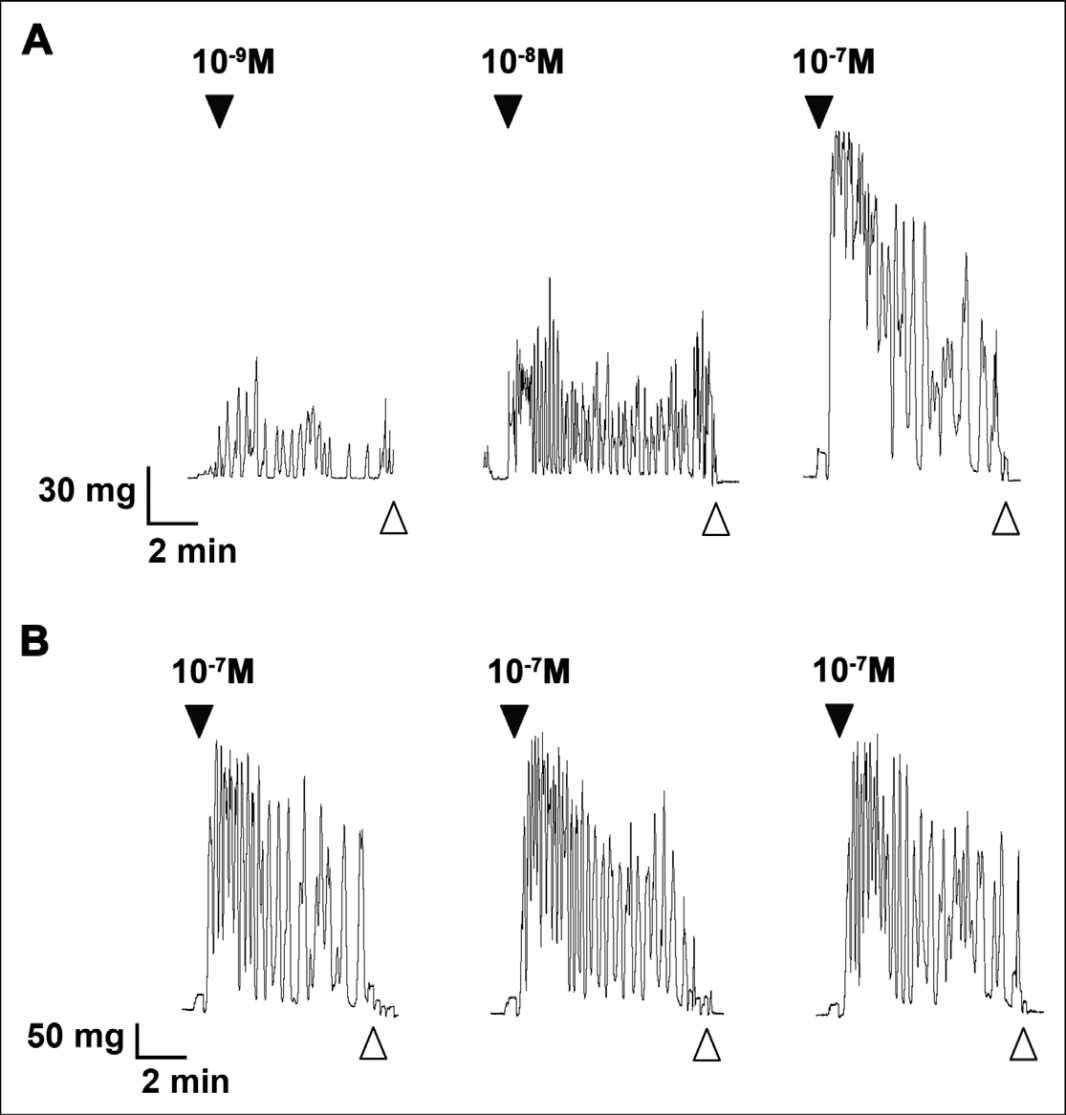
Leucokinin 1 (LK 1) induces contractions of *Rhodnius prolixus* hindgut incubated in physiological saline. (A) The effects of increasing doses of LK 1 on the phasic contractions and basal tonus of *R. prolixus* hindgut. At 10^-9^ M, LK 1 increases frequency of contractions but does not affect basal tonus of the preparation. However, increasing concentrations of LK 1 induced an increase in the frequency of phasic contractions, amplitude of contractions and a change in basal tonus. (B) Even at relatively high doses, LK 1 can induce increased myoactivity with repeated doses over time for up to 90 minutes. Closed arrows indicated addition of LK 1. Open arrows indicate washing with physiological saline. The preparations were washed at 5 minute intervals for 20 minutes between applications of peptide solutions.

**Figure 3.**
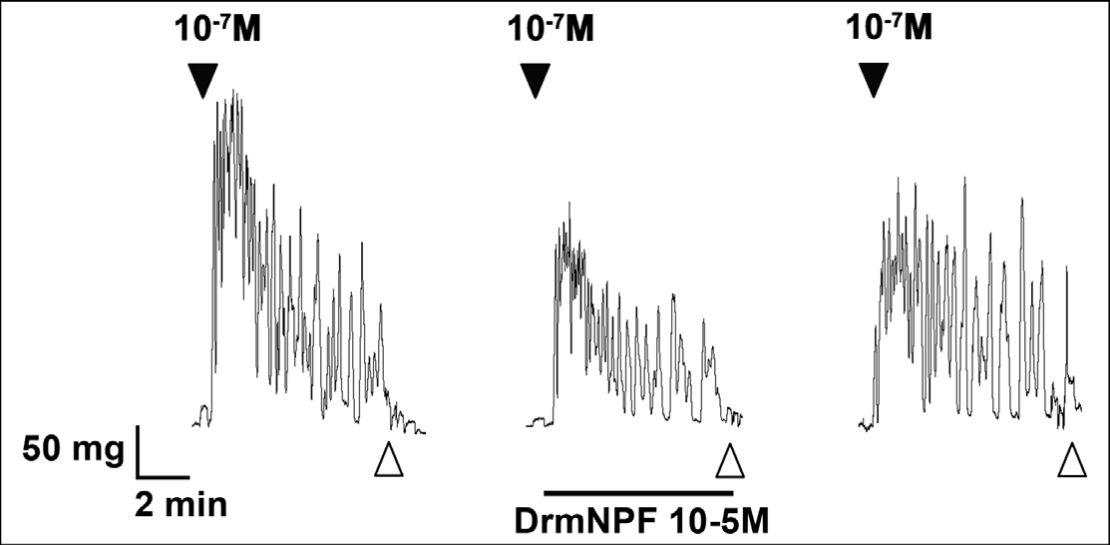
Effects of DrmNPF at 10^-5^M (horizontal bar) on leucokinin 1 (LK 1)-induced contractions (10^-7^M) of a single isolated *Rhodnius prolixus* hindgut. Closed arrows indicate addition of LK 1 at 10^-7^ M and open arrows indicate washing with physiological saline. Preparations were washed at 5 minute intervals for 20 minutes between additions of peptide solutions. The trace shown here is a representative trace of 3 preparations.

DrmNPF has an inhibitory effect upon the frequency and amplitude of spontaneous muscle contractions of the hindgut of 5^th^ instar *R. prolixus.* The inhibition of spontaneous hindgut contractions by DrmNPF is dose-dependent, with a threshold value at 10^-7^ M and complete inhibition at 10^-5^ M (Figure 1). There is, however, a lack of consistency in the amplitude and duration of spontaneous activity between preparations, and for a preparation repeatedly washed with saline or test solutions. Subsequently, in order to quantify the effects of DrmNPF and AngNPF more accurately on *R. prolixus* hindgut contractions, leucokinin 1 (LK 1) was used to induce a relatively consistent increase in hindgut muscle activity against which to challenge NPFs. When concentrations of LK 1 of 10^-9^ M and above are applied to the hindgut of *R. prolixus,* increases in contraction frequency and amplitude, as well as an increase in basal tonus are observed (Figure 2A). The activity of LK 1 is dose-dependent (Figure 2A), and robust with respect to repetitive applications (Figure 2B). When LK 1 at 10^-7^ M was challenged in the hindgut assay with DrmNPF (10^-5^ M), a decrease in basal tonus, frequency and amplitude of hindgut contractions was observed compared to LK 1 controls (N = 3) (Figure 3).

**Figure 4.**
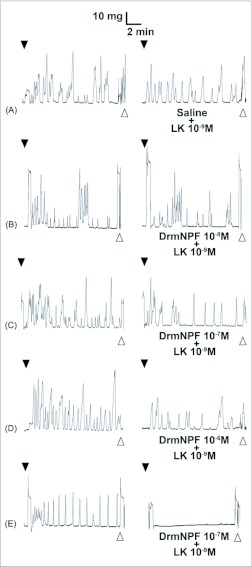
Effects of increasing concentrations of DrmNPF on leucokinin 1 (LK 1)-induced contractions of a single isolated *Rhodnius prolixus* hindgut. Closed arrows indicate addition of 10^-9^ M LK 1 (left column) or various concentrations of DrmNPF and 10^-9^ M LK 1 (right column). The addition of DrmNPF was always preceded by a trial of LK 1 alone (A–E). Open arrows indicate washing with physiological saline. The preparation was washed at 5 minute intervals for 20 minutes between applications of peptide solutions.

**Figure 5.**
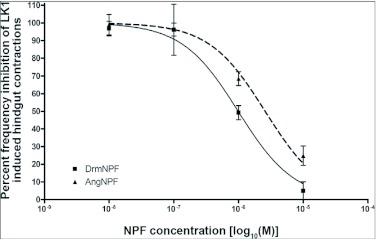
Dose-response curve showing the effects of DrmNPF and AngNPF on the frequency of leucokinin 1 (LK 1)-induced muscle contractions of isolated *Rhodnius prolixus* hindgut preparations. Results are expressed as a percentage of the frequency of muscle contractions stimulated by 10^-9^ M LK 1 alone. The average frequency was measured by force transducer for a 4 minute interval, 1 minute after the addition of the peptide solutions. Each point represents the mean ± SEM (N = 5).

**Figure 6.**
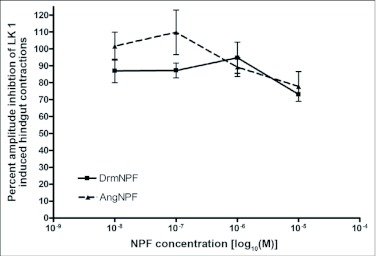
Dose-response curve showing the effects of DrmNPF and AngNPF on the amplitude of leucokinin 1 (LK 1)-induced muscle contractions of isolated *Rhodnius prolixus* hindgut preparations. Results are expressed as a percentage of the amplitude of muscle contractions stimulated by 10^-9^ M LK 1 alone. The average amplitude of contractions was measured for a 4 minute interval, 1 minute after the addition of the peptide solution. Each point represents the mean ± SEM (N = 5).

**Figure 7.**
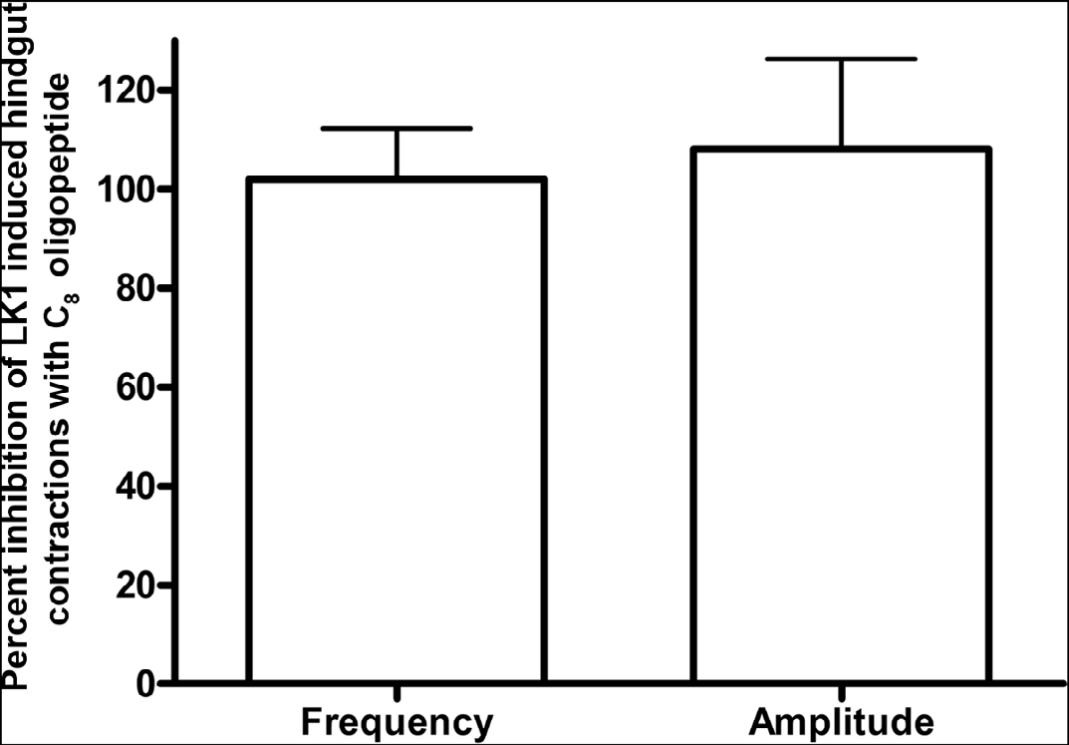
Effects of C_8_ oligopeptide (10^-4^ M) on leucokinin 1 (LK 1)-induced contractions (10^-9^ M) on *Rhodnius prolixus* hindgut. At 10^-4^ M, C_8_ had little or no effect on frequency or amplitude of contractions. Each bar represents the mean ± SEM (N =3).

When applied at a concentration of 10 M, LK 1 consistently induces an increase in contraction frequency and amplitude, with little change in basal tonus (Figures 2, 4). To quantify the inhibitory activity of NPFs, a concentration of 10^-9^ M LK 1 was chosen, and challenged with DrmNPF and AngNPF (Figures 4–6). DrmNPF inhibits the frequency of LK 1-induced contractions, with a threshold value of 10^-7^ M and an EC_50_ of 1 µM (N = 5) (Figures 4, 5). Complete elimination of contractions is observed in almost all preparations challenged with 10 DrmNPF (N = 5). The dose-response curve for AngNPF parallels that of DrmNPF, although AngNPF is slightly less effective with an average reduction in frequency of 75±12 % compared to controls when challenged with 10^-5^ M AngNPF (N = 5) (Figure 5). Interestingly, the NPFs only slightly inhibit the amplitude of LK 1-induced contractions (Figure 6). The C-terminal amidated oligopeptide, corresponding to the last eight amino acids of DrmNPF, C8 (GDRARVRFamide), at 10^-4^ M, had no inhibitory effect on hindgut muscle activity in the presence of LK1 (N = 3) (Figure 7).

### Effects of HPLC fractions of CNS on LK 1 -induced hindgut contractions

**Figure 8.**
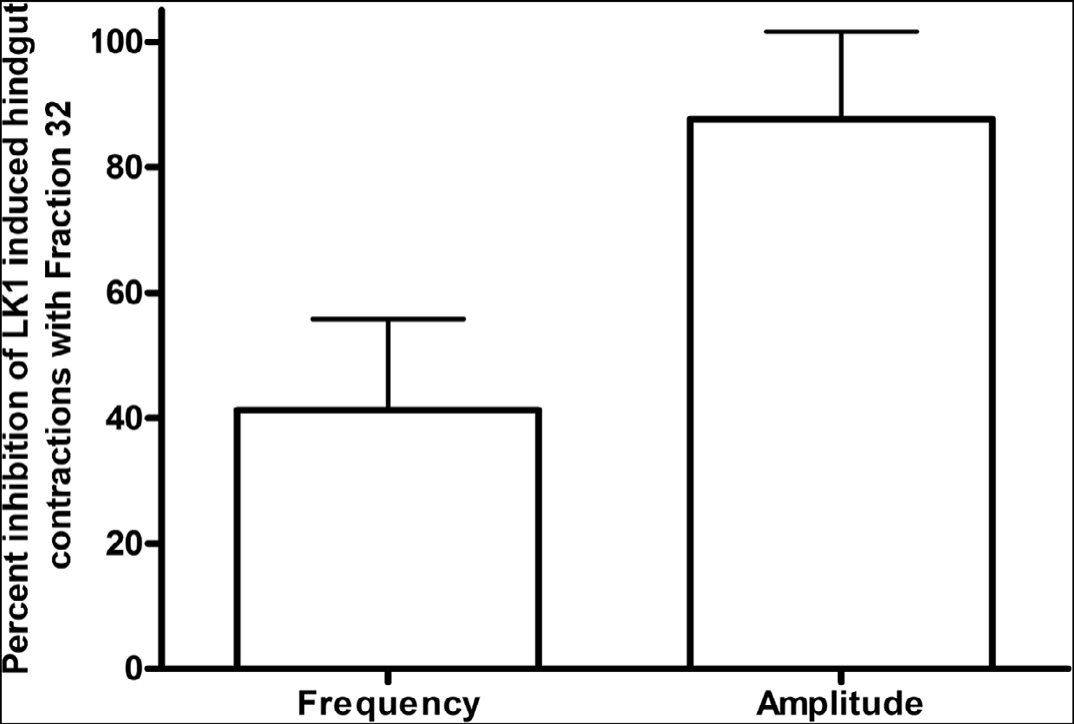
Effects of fraction 32 on leucokinin 1 (LK 1)-induced contractions of a single isolated *Rhodnius prolixus* hindgut. 3.3 CNS equivalents of fraction 32 inhibited the frequency of LK 1-induced contractions and has little effect on the amplitude of hindgut contractions. Each bar represents the mean ± SEM (N = 3).

**Figure 9.**
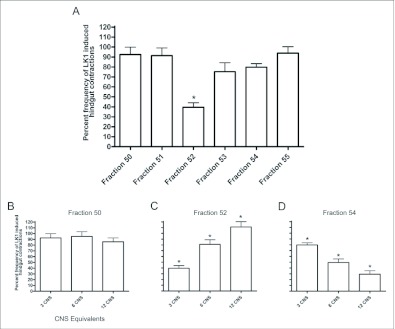
Effects of fractions 50–55 on leucokinin 1 (LK 1)-induced contractions of *Rhodnius prolixus* hindgut. (A) 3.3 CNS equivalents of fractions 50–55; fraction 52 significantly inhibited the frequency of LK 1-induced contractions. (B, C, D) Effects of increasing concentrations of fractions 50, 52, and 54 on LK 1-induced hindgut contractions. Each bar represents the mean ± SEM (N = 3, for each fraction). Statistics were performed on arcsine transformations. * p <0.05, oneway ANOVA Newman-Keuls post-test.

Hindgut assays provide positive testing for isolation of DrmNPF-like immunoreactive material collected from RP-HPLC fractionation followed with DrmNPF RIA (Gonzalez and Orchard 2008). For assay, 3.3 CNS equivalents of NPF-like immunoreactive material from RIA positive fraction 32, from the C_18_ column, were reconstituted in 270 µl of saline. Approximately 0.1 nM equivalents of NPF-like immunoreactive material from fraction 32 decreased the frequency of LK 1-induced contractions (10^-9^ M) by an average of 41 ± 15 % compared to controls, but had little effect on amplitude (N = 3) (Figure 8). Fractions 31–33, from the C_18_ column, were pooled and fractionated through a phenyl column using RP-HPLC (Gonzalez and Orchard 2008). NPF-like immunoreactivity was detected within fractions 50, 52 and 54. Fractions 50–55 were assayed for myoinhibitory activity (Figure 9). For assay, 3.3 CNS equivalents of fractions 50–55 were each reconstituted in 270 µl of saline and challenged against LK 1 (10^-9^ M). The frequency of LK 1-induced contractions was not reduced when applied simultaneously with fraction 50 (70 pM NPF equivalents) (N = 3). Similarly, fractions 51 and 55 had no effect on the frequency of contractions or amplitude (N = 3). Fraction 54 (70 pM NPF equivalents), and fraction 53 slightly reduced the frequency of LK 1-induced contractions from controls to an average of 80 ± 3 %, and 75 ± 16 % respectively, with no appreciable effect on amplitude (N = 3). Fraction 52 (40 pM NPF equivalents) significantly reduced the frequency of LK 1-induced contractions to an average of 40 ± 8 % (p <0.001) compared to controls (N = 3). Subsequently, more concentrated subsamples (3, 6, and 12 CNS equivalents) of fractions 50, 52, and 54 were assayed to challenge LK 1-induced contractions (Figure 9B–D). When higher doses of DrmNPF-like immunoreactive material from fraction 50 (0.15 nM, and 0.3 nM NPF equivalents, respectively) were applied, no change in frequency or amplitude of LK 1-induced contractions was observed (N = 3). Interestingly, the inhibitory influence of fraction 52 was less effective with increasing doses of DrmNPF-like immunoreactive material (0.8 nM and 1.6 nM NPF equivalents, respectively) (N = 3 for each dose). In contrast, an increased inhibitory influence of DrmNPF-like immunoreactive material from fraction 54 (49 ± 15 % and 31 ± 11 %, p < 0.05) was observed when preparations were challenged with higher doses (0.15 nM and 0.3 nM NPF equivalents, respectively) (N = 3 for each dose). The DrmNPF and AngNPF standards ran in fractions 54 and 51 respectively, through the phenyl column.

### Potassium ion fluxes across the hindgut epithelium *in vitro*

**Figure 10.**
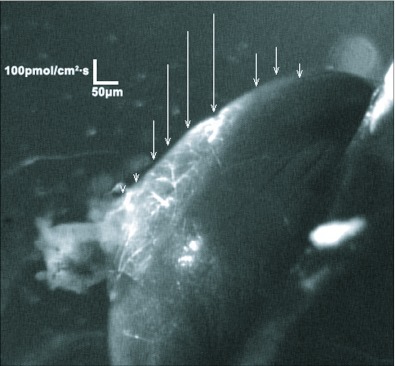
Representative example of SIET measurements showing calculated ion fluxes along the surface of the *in vitro* hindgut of *Rhodnius prolixus.* Localized ion flux was illustrated by vectors superimposed on a digital image of the hindgut. The direction of the vector reflects the movement of ions into (influx) or out of (efflux) the hindgut, whereas the length of the vector reflects the magnitude of the ion flux. Large influxes of K^+^ are observed over the ampulla that increased centrally and dissipated at the margins. No additional K^+^ fluxes were observed along the length of the hindgut. The lengths of the arrows indicate the magnitude and direction of the potassium flux (N = 10).

**Figure 11.**
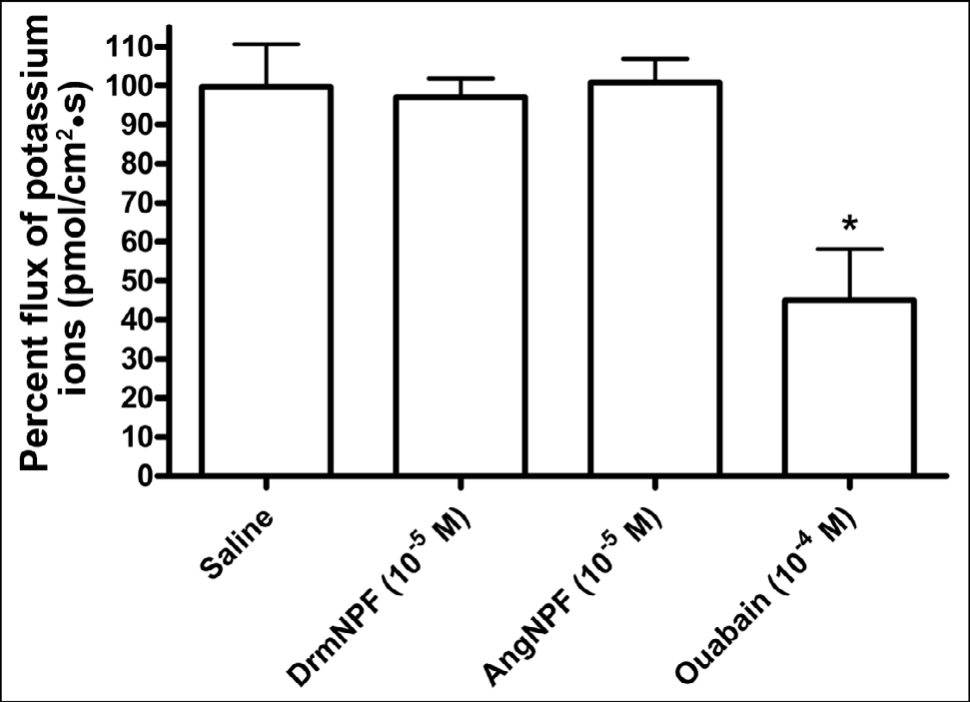
Potassium ion flux of a single isolated *Rhodnius prolixus* hindgut *in vitro* challenged with DrmNPF, AngNPF, and ouabain. The NPFs tested did not influence potassium ion transport across the ampulla of the hindgut. At 10^-4^ M ouabain significantly decreased potassium ion flux over the ampulla of the hindgut. Each bar represents the mean ± SEM (N = 3). Statistics were performed on arcsine transformations. * p < 0.05, one-way ANOVA Newman-Keuls post-test.

The flux estimates derived from the SIET measurements of K^+^ concentration gradients indicated a net influx (from bath to lumen) of K^+^ into the hindgut of unfed 5^th^ instar *R. prolixus.* Although the magnitude of the flux varied between different hindgut preparations (175 ± 17.5 pmol/cm^2^ sec), all of the preparations tested showed influx of K^+^ (N = 10). The SIET measures ion gradients at localized positions along the length of the hindgut allowing for an assessment of regional differences in the K^+^ influx (see Figure 10). Typical measurements of K^+^ flux along the length of the hindgut revealed a consistent pattern of increased K^+^ influx over the ampulla (Figure 10). However, there were no detectable measurements of K^+^ flux anywhere else along the length of the hindgut. The application of 10 µM DrmNPF or AngNPF to hindgut preparations did not alter K^+^ influx over the ampulla (N = 3) (Figure 11). In contrast, the application of sodium cyanide (1 mM) abolished this influx (not shown) and applications of ouabain (1mM) reduced K^+^ influx to 45 ± 13 % compared to controls (N = 3) (Figure 11). All preparations tested with ouabain showed only partial recovery of K^+^ influx when washed with saline.

## Discussion

Our previous work revealed NPF-like immunoreactive processes on the hindgut of 5^th^ instar *R. prolixus,* suggesting a potential neuroregulatory role for the putative native NPF (Gonzalez and Orchard 2008). A novel inhibitory influence for NPF is now shown on the hindgut for spontaneous and LK 1-induced hindgut contractions. Differences in the dose-response curves for DrmNPF and AngNPF may reflect differences in the peptides ability to stimulate the putative native NPF receptor. The application of C8 oligopeptide (GDRARVRFamide), corresponding to the last 8 C-terminal amino acids of DrmNPF, did not inhibit the frequency or amplitude of LK 1-induced hindgut contractions, suggesting that the N-terminal extension is important for receptor binding/ activation.

The possible existence of a putative *R. prolixus* NPF with myoinhibitory activities was studied by isolating DrmNPF-like immunoreactive positive fractions from RP-HPLC and testing them against LK 1-induced hindgut contractions. Initial purification with a C_18_ column led to the isolation of fraction 32 as a candidate (Gonzalez and Orchard 2008). The selection of fraction 32 for assay and further purification was based on the following: (1) the insect myosuppressin and other extended FLRFamides elute earlier on both C_18_ and phenyl columns under these conditions (Peeff, *et al.* 1994; unpublished observations), (2) the proximity of the elution time for the DrmNPF standard, (3) previous research which noted a myoinhibitory activity upon the phasic contractions of hindgut of 5th instar *R. prolixus* using CNS tissue extracts (Te Brugge et al. 2002), (4) RIA identification of DrmNPF-like immunoreactivity within fraction 32 from the C_18_ column, and (5) the inhibition of LK 1-induced hindgut contractions using subsamples of fraction 32. Thus, the evidence is suggestive of at least one NPF-related peptide within the CNS of *R. prolixus.* Further purification of pooled fractions 31–33 yielded 3 fractions from the phenyl column that contained DrmNPF-like immunoreactivity. The isolation of three fractions with DrmNPF-like immunoreactivity may be explained by the existence of several different post-translational forms of a single NPF-like peptide, the degradation of the full length NPF-like peptide, or the presence of distinct NPF-like peptides in the CNS of *R. prolixus.* Application of fraction 50 within the nanomolar equivalent range did not show any inhibitory action when challenged against LK 1-induced hindgut contractions. Fraction 52, although inhibitory on LK 1-induced contractions at relatively low equivalent doses (70 pM), was less effective at higher doses, suggesting desensitisation or co-elution of other stimulatory factors within the fraction. However, the most promising candidate for the putative native NPF peptide lies within fraction 54, the fraction co-migrating with the DrmNPF standard. The dose-dependent inhibition of LK 1-induced contractions, and the elution of NPF-like immunoreactive material from the CNS of *R. prolixus* in fraction 54, is suggestive of a native *R. prolixus* NPF which may be similar in structure to *Drosophila* NPF.

Previous studies have shown that NPF can decrease the transepithelial voltage across the anterior stomach of *A. aegypti* larvae (Onken et al. 2004). This effect is dose-dependent and reversible, implicating NPF in the modulation of ion transport. Interestingly, evidence for the role of NPY in the modulation of ion transport in vertebrates has been described in the small intestine of humans and the distal colon of rats (Holzer-Petsche et al. 1991 ; Strabel and Diener 1995). Initial findings provided strong evidence for active transport of K^+^ over the ampulla of the hindgut in unfed 5^th^ instar *R. prolixus.* However, repeated applications of high doses of DrmNPF and AngNPF to the hindgut had no effect on the influx of K^+^ observed. Interestingly, ouabain was able to significantly reduce the influx of potassium, suggesting that this influx is coupled with reuptake of sodium into the hemolymph via a Na^+^ ,K^+^ -ATPase.

In the hindgut of 5^th^ instar unfed *R. prolixus,* K^+^ ion fluxes were localized specifically over the ampulla by direct measurement of ion concentration gradients along the length of the hindgut. Flux estimates indicate net influx of K^+^ into the lumen of the hindgut. These data are contrary to previous findings in locusts where ion selective electrodes have been used to investigate transport processes in the locust hindgut (Hanrahan and Phillips 1982). In locust hindgut preparations, the transepithelial potential across hindgut is established by active Cl transport towards the hemolymph side, creating a driving force for passive K^+^ reuptake from the lumen (net flux of K^+^ is towards the hemolymph side) through ion channels. Therefore, the direction of K^+^ transport in locusts is different from that observed in unfed 5th instar *R. prolixus.* However, this may be explained in part by diet, as locusts feed on K^+^ - rich substances and must be able to excrete excess K^+^ while conserving Na^+^ (G. Coast, University of London, personal communication). In contrast, *R. prolixus* needs to initially eliminate the excess Na from the blood meal while conserving K^+^ . The recently fed 5 instar *R. prolixus* used in this study will eventually need to eliminate excess K^+^ derived from the breakdown of red blood cells, and although the Malpighian tubules are known to autonomously regulate hemolymph K^+^ concentration (Maddrell et al. 1993), there is still likely to be a role for the hindgut in determining the composition of its expelled contents. The pattern of K^+^ influx observed in this case suggests that the lower Malpighian tubules recover K^+^ that is secreted by the upper Malpighian tubules so effectively that excess K^+^ must be eliminated by transport into the hindgut lumen.

The importance of continued research into NPF modulation of invertebrate systems is clear since NPF is related to one of the most complicated and intensely studied neuropeptides in mammals. Previous vertebrate studies have implicated NPY as an inhibitor of gastrointestinal motility (Holzer et al. 1987) and epithelial transport in the small and large intestine (Cox et al. 1988). Therefore, the presence of NPFs in other phyla and the physiological stability of these related neuropeptides across vast evolutionary time periods, suggests important fundamental roles for this family of peptides.

## Abbreviations

**Aea NPF**: Aedes neuropeptide F,
**Aeg NPF**: Anopheles NPF,
**CNS**: central nervous system,
**DrmNPF**: Drosophila neuropeptide F, LK 1: leucokinin 1,
**NPY**: neuropeptide Y,
**RIA**: radioimmunoassay,
**SIET**: Scanning ion-selective electrode technique
